# Prize-Essays on Insanity

**Published:** 1826-07

**Authors:** 


### MENTAL ALIENATION.*

Some time ago the celebrated Esquirol offered a prize for the best Essay on Insanity, and seven memoirs on this difficult subject were presented. The jury or commission appointed by the founder of the prize to examine and judge of them, have reported on three of the candidate essays only, as being more strictly on the subject proposed for investigation. Of these three essays, one appears to be treated rather magisterially by these plenipotentiaries. "Two of the memoirs," say they, "are composed in the spirit of rational and experimental medicine;—the third, on the contrary, belongs to the *anonymous* school of those physicians (happily not numerous) who think that diseases may exist without the lesion of any organ, and who hesitate not to introduce metaphysics into medicine." It is on this last memoir, of the *anonymous* school, that they first report, and we shall claim the privilege of commenting on their report as we proceed.

I. The Memoir is entitled—"*On the Distinction between Idiopathic (Essential) Alienation of the Mind, and that which is Symptomatic of Cerebral Affections.*" Under the first class, the author places "alienation purely moral," including certain cases of monomania, or delusion on a single subject—nostalgia—erotomania—fits of jealousy—thirst of vengeance, &c. The plenipotentiaries are up in arms at the supposition that the slightest deviation from perfect sanity of thought, word, or action, can depend on any thing but a *lesion of the brain.* They do not allow for any obliquity of the intellect or mode of thinking, from the force of habit or education. Thus, if a man were to present himself to this jury and aver (what he really believed) that he every day saw a supernatural being in the air, and not only saw him, but heard him speak there, the jury must, in conformity with their doctrine, not only put him down as insane—at least, upon the above point—but they must also place this mental hallucination to the score of a corporeal or material disease of the brain. And yet, whole nations entertain ideas, and firmly believe in absurdities equal to, or greater than, *those* of

——— the poor Indian whose untutor'd mind,
*Sees* God in clouds, and *hears* him in the wind.

From this it will be evident that if every *erroneous idea* be considered as dependent on corporeal lesion, there is not a man on earth who has a sound brain. For our own parts, we are inclined to believe, with the author of the stigmatized essay, that certain *moral causes* will produce impressions on the thinking principle within us (whether that be an immaterial principle in the brain, or the brain itself) which lead to erroneous conclusions, constituting, to all intents and purposes, INSANITY; and yet, without any disease, or appreciable alteration in the material organ of thought. But these considerations are far too refined for the jury. "Let us," say they, "recollect that the object of medicine is

* Archives, October, 1825.

*man*, considered in the *material* part, of which his *double being* is composed." (Consideré dans la partie materielle dont son *double être* est composé.) The devil it is! Why, yes—if by *medicine* they mean *medicines*, such as jalap, calomel, or the like—certainly these have only to do with the *material part* of man; but if they mean to exclude man's *moral nature* from the study of medicine—and to exclude the physician from the employment or the suggestion of *moral remedies*, then we say that the jury are bad physicians, whatever they may be as pathologists, restricting the latter term to the mere study of morbid anatomy. Do they not know that the best way of restoring reason, even where the insanity is evidently dependent on corporeal disease, is to combine moral with physical treatment? But it may be said that, as the brain is unquestionably the organ of thought, so any derangement of thought must be the consequence of derangement in its instrument. We do not think so. A deranged musical instrument will, undoubtedly, bring forth bad music—but so will the best instrument in the world, under certain impressions of the fingers. Moral impressions may lead the imagination and the judgment astray, and yet the brain may be sound in structure. But if for *structure* we substitute the word *function*, then we acknowledge that it will be difficult, or impossible, to prove that insanity is ever unconnected with an *affection* of the brain—because *thought is* the function of that organ, and derangement of thought is synonymous with derangement of cerebral function. Still we think that, for all practical purposes, there is sufficient ground for this distinction of insanity into that which is produced by moral causes, and unaccompanied by any symptom of disease—and that which, however caused, is accompanied by the phenomena of disease while living, and the material traces of it on dissection.

If this distinction of our author (which we think very proper) has caused such offence to this *physical* jury, they acknowledge that the portion of his essay which treats of insanity, as connected with and dependent on corporeal disease, is of very meritorious construction, not only as regards the description, but the etiology, pathology, and treatment of the disease. They remark one thing, however, which certainly appears a little extraordinary, viz:—that, from the pathological conditions of the brain, in symptomatic insanity, he has carefully excluded the term *phlegmasia or inflammation*, for which he seems to them to have a veritable antipathy. We wish they had given us a more detailed analysis of this author's essay.

II. The second Memoir bears for title—"*Essay on the Nature of Mental Alienation*." The author asks—"are there any particular alterations found in the brains of the insane? If so, what is their nature? what connexion or correspondence have they with the symptoms? Such is the triple problem which the author proposes to solve. The memoir is naturally divided into three parts. The first turns, of course, on the pathological anatomy of the brain and its dependencies in mania. The principal alterations of the brain are, injection, turgescence, increase of firmness, and the reverse, or softening—ulceration, hypertrophy—atrophy—accidental productions—hæmorrhage, &c. To these may be added a peculiar appearance, noticed by Esquirol, especially in indurations of the encephalic mass, viz.—the existence of holes or cavities in it, resembling those which we see in Gruyere cheese. This author, contrary to the former writer, regards all these various deviations from a healthy appearance in the brain, as resulting from acute or chronic inflammation. He next adverts to the morbid anatomy of the bony and membranous coverings of the brain in insanity, tracing them all to the same cause. He relates some cases where the nerves, issuing from the brain, were diseased. The second part of the essay contains a succinct history of 37 cases, (under the head ENCEPHALITIS,) of acute delirium, acute mania, acute monomania, chronic ditto, simple dementia, complicated ditto. These are accompanied by observations which have, for object, to establish that the acute delirium, mania, monomania, and dementia, are neither more nor less than encephalitis in different degrees of intensity. The third division of the memoir is a kind of resumée, in which, after comparing the symptoms during life, with the appearances on dissection, he endeavours to demonstrate anew that the said symptoms and organic changes belong to phlegmasiæ of varying intensity:—concluding, 1$^{mo.}$ That, in the brains of those who die of insanity we shall always find changes of structure—2$^{ndo.}$ That these changes are the consequence of inflammation acute or chronic—3$^{tio.}$ That there exists a correspondence between the symptoms and the organic changes—and that the names of monomania, mania, &c. ought only to be employed as representing degrees and stages of encephalitis. From this it will appear that he answers his three questions, as originally proposed, in the affirmative. He appears to have been placed in circumstances favourable for the elucidation of his subject, as he avers that he is possessed of the particulars of more than one hundred cases of insane people, whom he had an opportunity of examining after death, and comparing the symptoms with the dissections.

Biassed, as the jury evidently is, in favour of insanity, being *always* a corporeal disease, they are unwilling to adopt this author's doctrine in its fullest extent—because, on a minute analysis of the histories which he has given in his memoir, they have not been able to observe that strict connexion between the symptoms and dissections on one hand, and the mental alienation on the other, which he has insisted on. They consider that, in many of his cases, encephalitis has appeared to have accidentally supervened on insanity, and was in no way connected with the cause of the disease. They remark also, that many of the cases are defective, in respect to important details, and that his reasonings upon them are often far from being strictly logical, or his deductions legitimate. His cases, it is true, are numerous; but, as Morgagni justly observed—" *non numerandæ sed perpendendæ sunt observationes.*" In short, they hesitate not to aver, that many of his cases are not at all cases of acute mania, as he would make them, but *bonâ fide* cases of encephalitis—a circumstance that introduces much confusion into a memoir, in many other respects, valuable.

III. The third Memoir is entitled—" *On Epilepsy, considered in its Relation with Mental Alienation.*" This is divided into four sections, the first exhibiting a table of the dissections of epileptics—of epileptic insane—of insane without epilepsy—and of patients affected with diseases of the brain approaching to epilepsy and to insanity. The second is on epileptic maniacs—the third on etiology—the fourth on the treatment of insanity.

The first section contains four cases, with dissections, of simple epilepsy—and 14 dissections of epilepsy complicated with insanity. Five cases, with dissections, happened under the eye of M. Esquirol himself, and being well authenticated, we deem it right to notice some of them here.

*Case* 1. Saillant, aged 20 years, had been subject to epilepsy for some time previous to the month of May, 1825, when she became insane. On the 26th of that month she had 10 or 15 violent paroxysms, succeeded by profound coma, startings, tetanic rigidity, and other formidable symptoms. In this state she lingered twenty days, and then sunk.

*Dissection.* Slight meningitis—turgescence of the cerebral vessels—thickening and induration of the cortical, as well as the medullary substance of the brain. In short, this was a fine example of *induration* of the brain.

*Case* 2. This was a female, aged 32 years, who was first seized with mania of a furious kind. The mania continued long, and she was several times discharged the hospital uncured. In two years it changed into dementia, with a monthly paroxysm of excitation, and afterwards epileptic convulsions, tetanic rigidity, &c. In one of these paroxysms she expired, on the 7th January, 1825. On dissection there was found meningitis, with effusion into the cavity of the arachnoid—great injection of the cerebral vessels, especially of the cortical substance, which was soft—while the medullary was indurated, injected, and full of red points when sliced.

These are specimens of the diseased appearances which our author found in the insane and epileptic. He remarks that, in 18 dissections of epilepsy and epileptic-mania, there were 11 instances of *induration* of the brain—four of *softening*—and three where the consistence of the brain was natural. He thinks that such a large proportion of the appearances above-mentioned cannot have been accidental, but must be viewed in the light of pathological causes of the living phenomena. His mode of explanation of these diseased conditions of the brain is more satisfactory to himself than it will be to others. Both induration and softening are of the same nature—both are produced from chronic inflammation. But how is this inflammation produced? "In a paroxysm of epilepsy," says he, "the blood rushes to the brain—the congestion, thus repeated often, establishes a centre of fluxion in the organ—and this centre of fluxion continuing, determines a kind of combination of the blood with the cerebral substance, by which its density is increased." Thus then, according to his own explanation, the induration or the softening of the brain is not the *cause* but the *effect* of epilepsy. And so we verily believe it is. In fact, we are disposed to think that the same observation will apply to almost all other diseases as well as to insanity. It is hardly possible that the *causes* of disease can be revealed on dissection. They are too evanescent for the scalpel. The *function* of a part must always be disordered before the *structure* is altered, and the causes of disordered function are invisible, or long fled before dissection can be performed. We shall endeavour to illustrate this. A man is exposed to wet and cold, and does not change his clothes—or he sleeps in damp sheets. In a day or two he is seized with pain in his side, and cough. Remedies prove unavailing, and, in a fortnight or three weeks, he dies. By dissection, we hope to find the cause and nature of the disease; we do find a collection of sero-purulent matter in one of the cavities of the pleura. Is this the cause of the disease? No. It is the cause of *death*, but not of the disease of which the man died. Cold and wet to the surface excited inflammation of the pleura—this inflammation terminated in effusion—and this effusion killed the patient. Dissection, therefore, can rarely reveal to us the causes of disease—but only the ultimate stage or condition of the disease itself, by which death is caused. Into how many errors have medical men been led by neglecting this distinction! Thus, they open a man who died of fever, and they find inflammation of the brain. Oh, say they, this inflammation was the cause of the fever—and with just the same reason that the French author makes hardening of the brain the cause of epilepsy, though he, in the same breath, accounts for the hardening by the repeated attacks of the disease!

As for the causes of insanity, we believe they are such as first disorder the *function* of the brain—thought. They may be either moral or physical—as misfortunes, grief, &c. on one hand; or as intoxication, disorders in different parts of the body, &c. on the other. Now where the causes are moral, how absurd would it be to treat the disease in the same way as where the causes are physical? Two men present themselves to a physician—both with mental alienation. The mental derangement is caused, in one case, by losses in trade, or by wild speculation—in the other, by intemperance, producing disease of the liver and digestive organs. If insanity consists in a certain pathological condition of the brain, we ought to put these two patients upon the same course. If we are not to look to man's moral nature, we have only calomel and sarsaparilla for the man who lost his fortune and his reason by speculation, as well as for the man whose liver and stomach are disordered from brandy, and whose brain is symptomatically affected from the chylopoietic derangement. Here we see the absurdity as well as the danger of not keeping our eye on the *morale* as well as the *physique*—and hence the utility in a practical point of view, at all events, of dividing insanity into mental and corporeal.

We do not deem it necessary to dwell on our author's *treatment* of epilepsy and insanity. We believe that, in this country, we are quite on a par with our neighbours, as to the treatment of mental, as well as corporeal diseases. It is true our Gallic brethren entertain a very mean opinion of us in therapeutics, in consequence of the energetic measures which we pursue in dangerous diseases, and of our entertaining opinions that certain medicinal agents have *specific* effects on the human frame. Hence they characterize our prescriptions, as polypharmaceutic and heroic—and our practice as completely empyrical. We would recommend charity to the profession on both sides of the channel. It was an observation of Celsus, or rather of the physicians in his time, for we do not think he practised medicine, that the people of different countries required different modes of treatment, even in the self-same disease. If our polypharmacy would poison Frenchmen, their slip-slop treatment would murder Englishmen—hence, perhaps the account is pretty nearly balanced between them.

We must now draw to a close. We forgot, however, to mention that the author of this last memoir comes to the conclusion, (and in this conclusion he is supported by the decision of the jury,) that, in epilepsy, the seat of the disease is in the *medullary* portion of the brain, whilst that of insanity is in the *cortical.* This is also the opinion of Foville, Delage, and Pinel Grandchamp. We shall introduce the following short extract, as perhaps not quite unworthy of attention in a practical point of view.

"There exists a principle in physiology which may serve as a basis for the treatment of insanity, viz. that the more an organ is exercised, the more it becomes developed, the more it abounds with excitability—and the less are other organs developed and supplied with excitability. Hence it is necessary, in treating insanity, to leave in absolute repose the disordered encephalon, and augment the activity of other organs in the system." The jury cite a fact in support of this principle, namely, that all epileptics, on coming into hospital, experience a great diminution in the force and frequency of the paroxysms. In their new abodes they have few or no cares—no wants to provide for, or think about—and their brains sink, as it were, into a state of absolute repose, as compared with their former condition. After a time they begin to think and to become disquieted, and then the epileptic paroxysms return with their accustomed force.

Much as the jury applaud this last writer, they cannot coincide with him upon all points. They are disposed to admit that epilepsy is always occasioned by simple inflammation of the brain. The suddenness of its attacks, the occasional length of its intermissions, and the perfect integrity of intellectual functions during those intervals, in numerous cases, demonstrate the absurdity of this exclusive doctrine. As a resumée, they observe that the general results to be gathered from these memoirs are as follow:—1^mo.^ Mental alienation leaves in the brain traces of its existence, almost constant. 2^ndo.^ The traces left by insanity, or the alterations discoverable in the brain after death, are generally those which characterize acute or chronic inflammation. 3^tio.^ These alterations in the brain produce symptoms differing according as the change is in the cortical or the medullary substance: thus slight lesions of the superficial cortical structure are announced by disturbance of the *intellect*—by an alienation of the understanding—whilst lesions of the medullary portion of the brain are characterized by disturbance of the moving powers, to wit, by epileptic affections. But such is the intimate connexion between the cortical and cineritious substances of the brain, that lesions of the one are soon propagated to the other—hence the frequency of insanity and epilepsy in combination or in succession.

This is a very pretty theory on paper; but we much doubt whether it will stand the test of accurate clinical observation, and minute *post mortem* investigation. Such as it is, reader, we give it unto thee.

